# Using Isolated Femoral Bifurcation Endarterectomy or Combined with Bypass Surgery for Patients with Chronic Limb-Threatening Ischemia

**DOI:** 10.3390/medicina60020316

**Published:** 2024-02-12

**Authors:** Edin Ahmic, Wolfgang Hitzl, Rainald Seitelberger, Klaus Linni

**Affiliations:** 1 Department of Cardiovascular and Endovascular Surgery, Paracelsus Medical University Salzburg, Müllner Hauptstrasse 48, A-5020 Salzburg, Austria; r.seitelberger@salk.at (R.S.); k.linni@salk.at (K.L.); 2 Research Office (Biostatistics), Paracelsus Medical University, Müllner Hauptstrasse 48, A-5020 Salzburg, Austria; wolfgang.hitzl@pmu.ac.at

**Keywords:** endarterectomy, bypass surgery, femoral artery, lesion

## Abstract

*Background and Objectives*: The aim of this study was to evaluate the clinical outcomes of patients suffering from chronic limb-threatening ischemia (CLTI) and tissue loss treated with primary isolated femoral bifurcation endarterectomy (FBE) or with FBE combined with bypass surgery. *Materials and Methods*: This retrospective study was performed in a tertiary university-based care centre. Between January 2008 and December 2019, a prospectively collected database of patients suffering from CLTI and tissue loss and undergoing either primary FBE (group A) or FBE in combination with bypass surgery (group B) was analysed. Study endpoints were ulcer healing, primary and secondary patency rate, limb salvage, and survival. *Results*: In total, FBE was performed in 73 patients and FBE with bypass in 60 patients. Between both groups, there were no significant differences regarding demographic data or the Global Limb Anatomic Staging System (GLASS) grade III and IV of femoropopliteal lesions. After 3 years, ulcer healing could be achieved in 72% of FBE and in 75% of FBE with bypass patients. The primary patency rate was 95% and 91% for FBE and 83% and 80% for FBE with bypass after one and three years, respectively. The 3-year limb-salvage rate was 78% for FBE and 84% for FBE with bypass. The secondary patency rate after one and three years was 99% and 97% for FBE and 93% and 88% for FBE with bypass. *Conclusions*: FBE and FBE with bypass are equally effective for ulcer healing in cases of combined CFA and superficial femoral artery lesions. There was no significant difference between both groups regarding primary and secondary patency rates, limb salvage rates and ulcer healing. Isolated FBE could be an alternative strategy in patients with higher operative risk.

## 1. Introduction

Chronic limb-threatening ischemia (CLTI) with tissue loss is a serious medical condition that may have a wide range of severe consequences, and can lead to major amputation or death [1]. CLTI is a clinical syndrome defined by the presence of the peripheral artery disease combined with rest pain, gangrene, or a lower-limb ulceration of >2 weeks duration [2]. The gold standard for the treatment of patients with CLTI due to lesions of the common femoral artery (CFA) and its bifurcation is femoral bifurcation endarterectomy (FBE) [3,4,5,6,7,8]. In cases of CFA lesions combined with long superficial femoral artery (SFA) lesions, data regarding the best treatment are scarce [9]. Some authors preferred primary isolated FBE [10], whereas others advocated FBE combined with bypass surgery [9]. Now, the current guidelines recommend in-line flow via a targeted arterial pathway for optimal treatment of CLTI [2]. This retrospective study aimed to evaluate the clinical outcomes of patients with CLTI and tissue loss treated with primary isolated FBE or FBE with bypass.

## 2. Material and Methods

### 2.1. Study Design

This retrospective study was conducted at a tertiary university-based care centre. A prospectively collected database of patients with CLTI and tissue loss who underwent primary isolated FBE (Group A) or primary FBE combined with bypass surgery (Group B) was analysed. The local ethics committee approved this study.

Patients with Global Limb Anatomic Staging System (GLASS) [2] grade III and IV of femoropopliteal lesions who underwent primary isolated FBE or primary FBE combined with primary autologous vein surgical bypass (location below or above the knee) presented with intermediate or advanced limb-threatening conditions. The exclusion criteria were autologous bypass reconstruction combined with FBE to facilitate proximal anastomosis, composite or synthetic bypass grafts, additional intraoperative transluminal angioplasty, and occluded deep femoral artery (DFA). We have excluded all DFA occlusions, which could not be treated endovascularly or with open surgery so that all patients included in our study had a patent DFA. Patients suffering from mixed or venous ulcers were also excluded.

The primary endpoint of this study was ulcer healing. The secondary endpoints were primary and secondary patency rates, limb salvage, and survival. All patients underwent preoperative peripheral vascular evaluation with physical examination, oscillography, and ankle brachial index (ABI) measurements. Vascular lesions were confirmed using duplex sonography (DS), computed tomography angiography (CTA), magnetic resonance angiography (MRA), and selective angiography. FBE alone or combined with bypass reconstruction was performed at the surgeon’s discretion. All procedures were performed under either general or epidural anaesthesia.

### 2.2. Definitions

Ulcer healing was defined as the complete epithelialisation of the tissue defect. Ulcers were considered non-healed if they failed to heal or in cases of major amputation. In patients who died before the lesion epithelialized, the ulcer was considered non-healed, with the date of death as the cut-off point.

Ulcers were categorised according to the Wound, Ischemia, and Foot Infection (WIfI) classifications for peripheral arterial disease [2].

Major amputation refers to amputation above the ankle. The limbs that required major amputation were not salvaged. Limbs that required minor amputation (toe, ray, or trans metatarsal) and healed were considered successful limb salvage.

Primary patency was defined as the period free from reintervention to maintain patency. Secondary patency was defined as the time from the first intervention to restoration of patency after occlusion. For hemodynamic improvement, the ankle brachial index (ABI) should increase to ≥0.10 or to an ABI of ≥0.9.

Repeat target lesion reintervention (TLR) was measured with the frequency of the need to redo procedures due to a problem arising from the lesion, and repeat target extremity revascularization (TER) measured with the frequency of the need to redo procedures due to a problem arising remote from the lesion initially treated.

### 2.3. Lesion Anatomy

Lesions (occlusion or high-grade stenosis >70%) of the femoral bifurcation were divided into isolated CFA, combined CFA/proximal SFA, combined CFA/DFA, and combined CFA/DFA/proximal SFA.

### 2.4. Operative Technique

FBE and bypass procedures were performed under either general or regional anaesthesia. After preparation and exposure to CFA, 5000 IU of unfractionated heparin were administered. After clamping the femoral bifurcation, the CFA was incised, and endarterectomy was performed with Dacron or autologous patch graft angioplasty.

In cases of additional bypass surgery, veins were used as the graft material. The veins were evaluated using preoperative DS. Venous grafts included the greater saphenous, smaller saphenous, and arm veins. The grafts were used in reverse or non-reverse configurations after valve lysis.

After endarterectomy of the groin vessels, proximal anastomosis was performed with 6/0 Prolene using the continuous suture technique. Bypasses were placed either anatomically or subcutaneously.

The distal anastomosis was reconstructed in an end-to-side or end-to-end fashion. Complete angiography was performed to ensure an adequate graft flow.

### 2.5. Follow Up

Patients were continually monitored during the ulcer healing process in an outpatient setting. Additionally, all patients were followed up at 1 week and, 1, 3, 6, and 12 months postoperatively. The attending surgeon evaluated the ulcer status and local management. Ulcer status and the time to complete healing were recorded. In cases of wound worsening or infection, antibiotic therapy was started or adapted, and the vessel status was re-evaluated using oscillography and DS, and, if necessary, using CTA, MRA, or selective angiography.

The postoperative ulcer status was defined as stable, healed, or worse. The stable wounds were continuously monitored and bandaged. The healed wounds were completely epithelialized and no further dressing was required. Deteriorating wounds required antibiotic treatment and further re-evaluation of circulation (see above).

### 2.6. Statistical Analysis

Data consistency was checked and the data were screened for outliers. Continuous variables were also tested for normality using the Kolmogorov–Smirnov test. Cross-tabulation tables with Pearson’s chi-squared and Fisher’s exact tests were used to compare discrete variables, and *t*-tests were used for continuously distributed variables. Kaplan–Meier analyses with log-rank tests were used to compare the FBE and FBE + bypass groups. Pointwise 95% confidence intervals (CI) for the time-to-event curves were computed. All reported tests were two-sided, and *p*-values <0.05 were considered statistically significant. All statistical analyses were performed using STATISTICA 13 (Hill, T. & Lewicki, P. Statistics: Methods and Applications. StatSoft, Tulsa, OK, USA) and NCSS (NCSS 10, NCSS, LLC. Kaysville, UT, USA).

## 3. Results

A total of 133 patients who underwent surgery between 01/2008 and 12/2019 were included in this study. FBE (group A) was performed in 73 patients, and FBE and bypass (group B) in 60 patients. All 133 patients received duplex ultrasound (100%) in both groups, 105 patients (79%) received CTA, and 28 patients (21%) received MRA, respectively. Additionally, the selective arteriography was performed in 18 patients (13%). Thirteen (21.6%) patients in group B received bypass above the knee, and bypass below the knee was performed in 47 (78.3%) patients. There were no significant differences between the groups in terms of demographic data, risk factors, comorbidities, and preoperative ABI (Table 1). Wound and foot infection grading in WIfI classification was equally distributed. There was also no significant difference between groups A and B regarding GLASS classification femoropopliteal or infrapopliteal, lesion anatomy of the femoral bifurcation (Table 2). CFA occlusions were equally distributed between group A and B (*n* = 28 vs. *n* = 24, 38% vs. 40%, *p* = 0.78) (Table 2). There was no significant difference regarding the mean length of CFA lesions, (2.65 cm vs. 2.52 cm, *p*= 0.12) and no significant difference regarding the mean diameter of treated CFA lesions (6.48 vs.6.3 mm *p* = 0.41). The mean diameter of DFA was equal in both groups 5.75 vs.5.28 mm (*p* = 0.57).

The median follow-up time was 2.7 years (lower quartile: 1.38, upper quartile 4.56). It was completed by all patients, and none of them were lost to follow-up.

**Figure 1:**
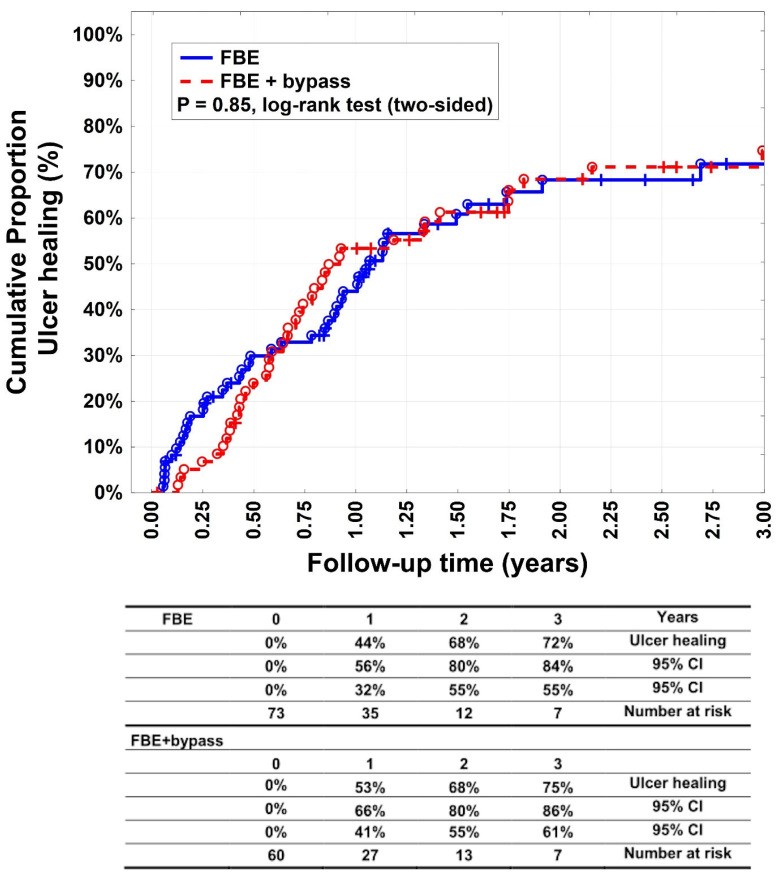
Ulcer healing. Kaplan–Meier estimates of wound healing, comparing patients undergoing femoral bifurcation endarterectomy (FBE) or FBE with additional bypass (FBE + bypass).

There was no statistically significant difference (*p* = 0.85) between groups A and B with respect to complete ulcer healing (Figure 1). Complete ulcer healing was achieved in 44% (95% CI: 32–56%), 68% (95% CI: 55–80%), and 72% (95% CI: 55–84%) of patients in Group A after 1, 2, and 3 years, respectively. In Group B, complete healing was observed in 53% (95% CI: 41–66%), 68% (95% CI: 55–80%), and 75% (95% CI: 61–86%) of patients after 1, 2, and 3 years, respectively.

**Figure 2:**
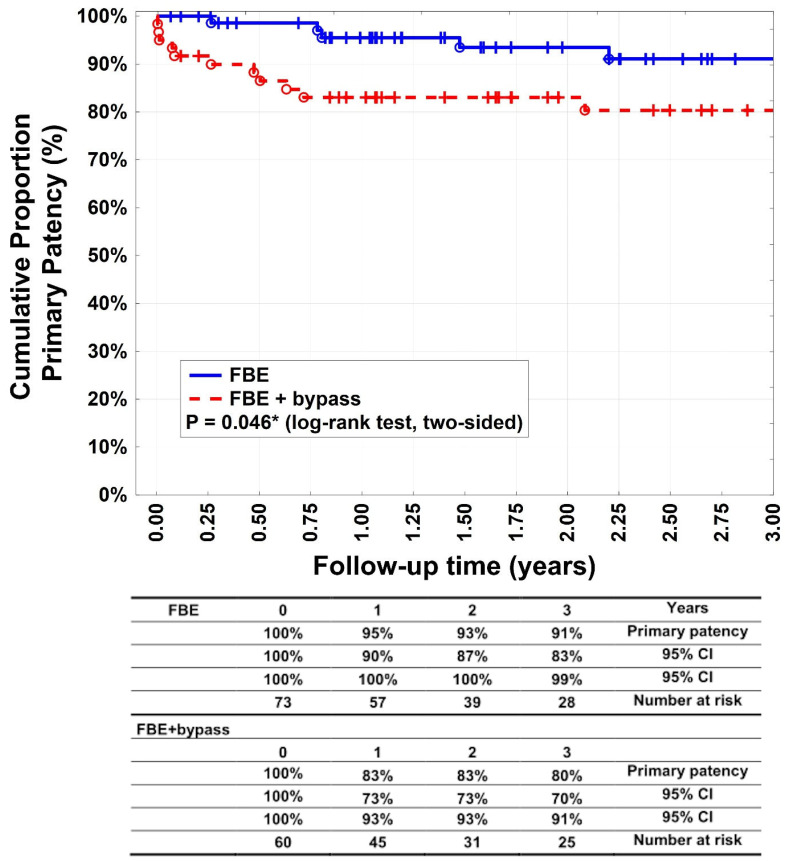
Primary patency. Kaplan–Meier estimates of primary patency, comparing patients undergoing femoral bifurcation endarterctomy (FBE) or FBE with additional bypass (FBE + bypass).

**Figure 3:**
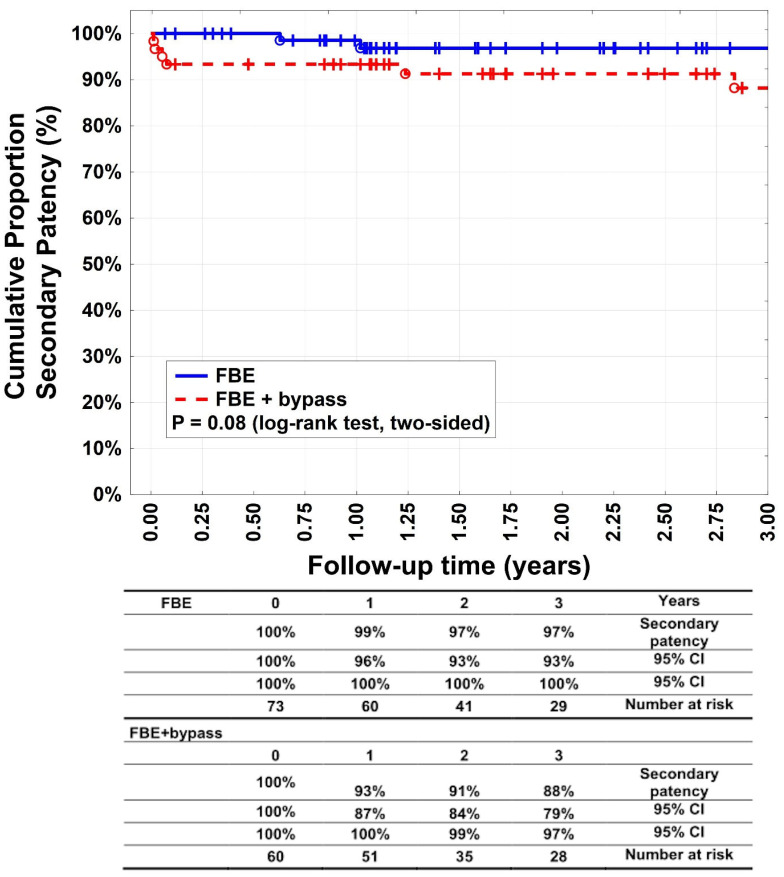
Secondary patency. Kaplan–Meier estimates of secondary patency, comparing patients undergoing femoral bifurcation endarterectomy (FBE) or FBE with additional bypass (FBE + bypass).

The primary patency rates (Figure 2) after 1, 2, and 3 years were 95% (95% CI: 90–100%), 93% (95% CI: 87–100%), and 91% (95% CI: 83–99%), respectively, in group A patients. For group B, they were 83% (95% CI: 73–93%), 83% (95% CI: 73–93%), and 80% (95% CI: 70–91%), respectively (*p* = 0.046%). Secondary patency rates (Figure 3) were 99% (95% CI: 96–100%), 97% (95% CI: 93–100%), and 97% (95% CI: 93–100%) after 1, 2, and 3 years, respectively, for patients in group A. For group B, they were 93% (95% CI: 87–100%), 91% (95% CI: 84–99%), and 88% (95% CI: 79–97%) after 1, 2, and 3 years, respectively (*p* = 0.08). Target lesion reinterventions were necessary in four patients (5.5%) in group A and for 13 patients (21.6%) in group B (*p* = 0.0001). Each of the subgroups (bypass below knee and bypass above knee) in group B had 4 and 9 patients who had target lesion reinterventions as well as 18 and 5 patients with target extremity revascularisation.

The mean postoperative ABI was 0.71 ± 0.33 in Group A and 0.82 ± 0.2 in group B. This difference was statistically significant in interim analysis (*p* = 0.001).

We observed 11 (15%) surgical-site infections (SSIs) in Group A patients and 12 (20%) SSIs in Group B. This was not statistically significant (*p* = 0.42). All SSIs were minor and could be treated conservatively.

**Figure 4:**
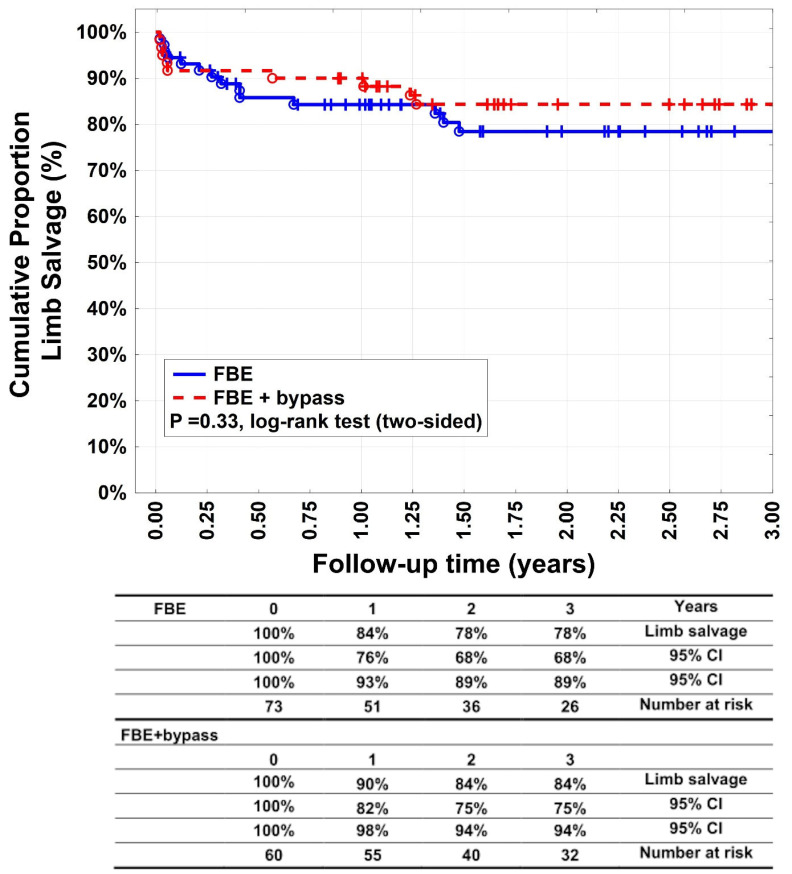
Limb salvage. Kaplan–Meier estimates of limb salvage, comparing patients undergoing femoral bifurcation endarterectomy (FBE) or FBE with additional bypass (FBE + bypass).

**Figure 5:**
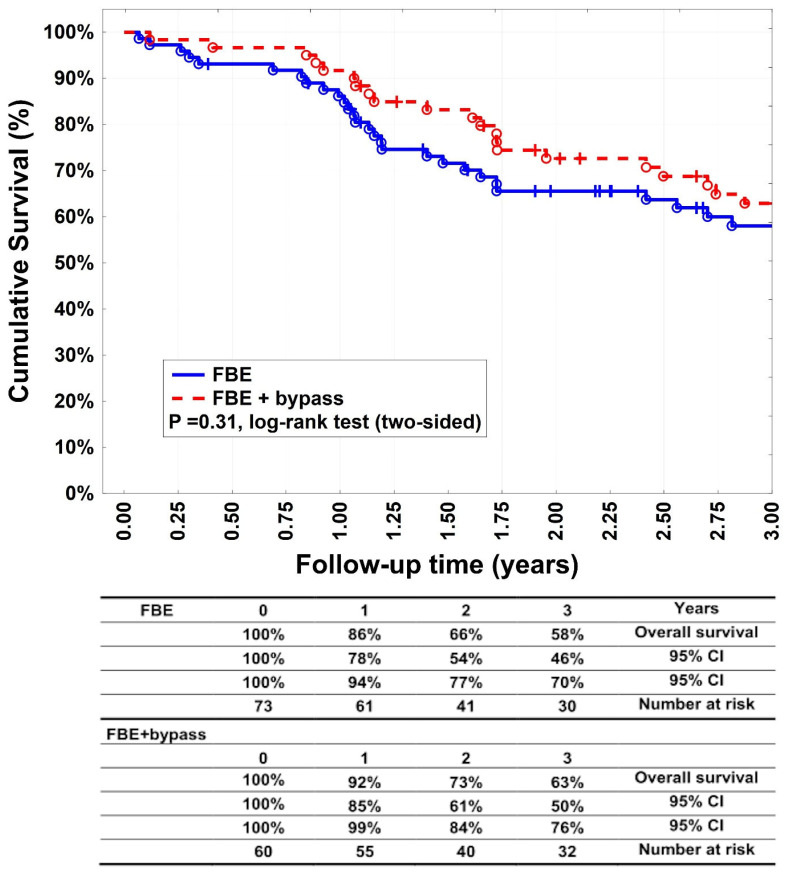
Cumulative survival. Kaplan–Meier estimates of cumulative survival, comparing patients undergoing femoral bifurcation endarteretomy (FBE) or FBE with additional bypass (FBE + bypass).

Target extremity revascularisation was indicated in 33 patients (45.2%) in group A and 23 patients (38.3%) in group B (*p* = 0.48). Twelve patients in group A (16.4%) of thirty-three, which needed reintervention, needed an additional bypass surgery. Limb salvage rates (Figure 4) after 1, 2, and 3 years were 84% (95% CI: 76–93%), 78% (95% CI: 68–89%), and 78% (95% CI: 68–89%) for group A patients and 90% (95% CI: 82–98%), 84% (95% CI: 75–94%), and 84% (95% CI: 75–94%) for group B patients (*p* = 0.33). Fourteen patients (19.2%) underwent major amputation in group A, versus nine patients (15%) in group B (*p* = 0.64). Group B had 47 patients with bypass below the knee and 13 patients with bypass above the knee. Each of these subgroups had 4 and 5 patients with major amputation as well as 15 and 13 patients with minor amputation, respectively. Twenty patients (27.4%) underwent minor amputation in group A, and 18 (30%) underwent it in group B (*p* = 0.84). Survival rates (Figure 5) were 86% (95% CI: 78–94%), 66% (95% CI: 54–77%), and 58% (95% CI: 46–70%) after 1, 2, and 3 years, respectively, in group A. In group B, they were 92% (95% CI: 85–99%), 73% (95% CI: 61–84%), and 63% (95% CI: 50–76%) after 1, 2, and 3 years, respectively (*p* = 0.31). Most patients died of cardiovascular events.

## 4. Discussion

This retrospective study showed no differences in clinical outcomes (ulcer healing and major amputation rate); however, there was a significant difference in the primary patency rates and target lesion interventions in patients with tissue loss due to CLTI who underwent either primary isolated FBE or FBE combined with bypass surgery.

Currently, evidence regarding the preferential use of FBE alone or combined with bypass surgery in patients’ CFA lesions and long-segment SFA occlusion is lacking [9]. Both methods are appropriate, and it is possible to choose one technique over another.

Malgor et al. compared isolated FBE and FBE combined with bypass surgery. In their retrospective study, which included Rutherford 3–6 patients with TASC A–D lesions, the authors found that patients with tissue loss and TASC D lesions benefitted from additional bypass procedures and that a low runoff score resulted in poor clinical outcomes [11]. However, the populations in the latter study were not equally distributed (claudicants were more common in the FBE group). According to Zlatanovic et al., the femoropopliteal GLASS IV grade is similar to that of TASC D [12]. In contrast, our study revealed no significant difference in patient characteristics or risk factors between groups A and B. Additionally, only patients with GLASS grade III and IV were observed. These patients are usually recommended to undergo open surgery [2,13].

A study performed by Soden et al. compared the 1-year outcomes of patients who underwent bypass surgery and additional FBE in lower-extremity bypass. In their retrospective study, the authors compared patients who underwent lower-extremity bypass with FBE with those who underwent bypass alone. They demonstrated that patients who underwent an additional FBE with bypass had improved 1-year freedom from major amputation compared with those who underwent bypass only (91% vs. 87%). Their results confirmed that additional FBE improves limb perfusion through the DFA and its collaterals, which is cause for a better outcome [9].

The primary endpoint of our study was ulcer healing, which did not differ significantly between groups A and B (*p* = 0.85). After 3 years, ulcers completely healed in more than 70% of patients in groups A and B, and there was no statistical difference in major amputations (*p* = 0.64) or minor amputations (*p* = 0.84). Furuyama et al. reported a median ulcer healing time of 90 days after bypass revascularisation or endovascular treatment. In their retrospective study, they described the prognostic factors after arterial infrainguinal revascularisation for Rutherford class 5 critical limb ischaemia and assessed the efficacy of cilostazol therapy. Complete ulcer healing was achieved in 74% of patients, which is similar to our results [14]. In their study, only patients with Rutherford class 5 were included, whereas we included patients with all ulcer categories according to the WIfI classification [2,15]. In our study, isolated FBE was as successful as FBE combined with bypass in terms of ulcer healing.

Our study showed a significant difference between groups A and B in terms of primary patency rates after 1, 2, and 3 years (*p* = 0.046), whereas the secondary patency rates were equal. Primary and secondary patency rates of > 90% in patients undergoing FBE have been previously reported and are consistent with our results [16,17]. Surprisingly, secondary patency rates did not differ significantly between the groups and reached 88% in the bypass group, whereas the literature revealed that secondary patency rates of isolated autologous bypasses were worse [11,18,19]. It is possible that obligatory FBE can enhance the bypass patency. Long-term patency rates after FBE with additional bypass revascularisation have rarely been reported.

El-Bakr et al. presented in their abstract at the IAVS & NIVASC Joint Annual Meeting in 2015 that for patients with TASC D lesions, FBE and profundoplasty were sufficient to maintain adequate limb perfusion. In their study, high-risk patients presenting with ASA grade ≥ 3 with femoropopliteal TASC-D lesions were considered for limited FBE endarterectomy with profundoplasty without further revascularisation. They found that limb perfusion through the DFA is effective and could be a safe option in patients in a poor condition [20].

In our study, patients in group B underwent significantly more target lesion reinterventions than those in group A patients (23% vs. 5.5%, *p* = 0.0001). Balotta et al. described freedom from revascularization after FBE of the ipsilateral extremity as 80% after 7 years [16]. Kim et al. described endovascular femoropopliteal bypass reintervention rates of up to 21% after 1-year [21]. In another retrospective study, the reintervention rate for bypass was 30% during a 5-year follow-up period [12]. The target extremity reinterventions were not significantly different between the groups in our study (*p* = 0.48).

According to the recent literature, it is unclear whether a patent DFA combined with an occluded SFA is sufficient for adequate ulcer healing in patients with CLTI. De Athayde Soares et al. showed that PTA of the DFA alone shows a similar limb salvage rate, patency rates, and survival, as patients undergoing PTA of the DFA and SFA [22]. Our study showed that a patent DFA combined with SFA lesions was sufficient for adequate limb vascularisation.

Manenti et al. explained in their article on “the pathophysiology of the profunda femoris artery in chronic lower limb ischemia” that the DFA forms the main collateral pathway for the perfusion of the distal limb. According to these studies, in cases of chronic limb ischaemia, the DFA can progressively double in size because it is an elastic blood vessel. This may have been responsible for the formation of new collaterals in the DFA and its branches [23].

Studies evaluating the revascularisation effect before and after FBE are limited. The extent to which collaterals that enable sufficient healing of the ulcer are built after FBE when the DFA is patent and SFA is occluded. Measuring and counting collaterals before and after FBE using angiography may be useful in prospective randomised studies.

This study is limited by its retrospective design. The choice of revascularisation type was operator dependent. Randomised trials are required to determine the best approach for treating CLTI with tissue loss.

## 5. Conclusions

FBE is a less-invasive option for surgical repair of GLASS III and IV grade in patients with CLTI with tissue loss. It is a feasible option with good short- and mid-term results in patients with patent DFA. With the exception of primary patency, there was no significant difference in secondary patency, limb salvage, or survival rates between FBE and FBE with additional bypass. Our study suggests that FBE alone may be sufficient to achieve ulcer healing in a typically frail, elderly population and avoids the potential morbidity, and necessary for venous conduit and prolonged surgery associated with distal bypass. As a less-invasive procedure, FBE should be considered first for patients in a poor general condition.

## Figures and Tables

**Table 1:** Patient characteristics, risk factors, and comorbidities.

	Group A (FBE, *n* = 73)	Group B (FBE + bypass, *n* = 60)	*p*-Value
Age (mean)	76	74.5	0.39
Male/female	47 (64.4%)/26 (35.6%)	39 (65%)/21 (35%)	1.0
BMI	25.42	25.26	0.84
Arterial hypertension	69 (94.5%)	51 (85%)	0.082
Dyslipidemia	41 (56.2%)	39 (65%)	0.37
Diabetes mellitus	28 (38.4%)	27 (45%)	0.48
Coronary heart disease	35 (47.96%)	22 (36.7%)	0.22
Renal insufficiency	25 (34.3%)	15 (25%)	0.26
Dialysis	6 (8.2%)	6 (10%)	0.76
Atrial fibrillation	21 (28.8%)	16 (26.7%)	0.84
Current smoker	25 (34.3%)	22 (36.7%)	0.85
Former smoker	16 (22%)	18 (30%)	0.32
Previous ipsilateral PTA	17 (23.3%)	22 (36.7%)	0.12
Mean preoperative ABI	0.46 (SD ± 0.15)	0.42 (SD ± 0.21)	0.051
Graft location			
Bypass above knee		13 (21.6%)	
Bypass below knee		47 (78.3%)	

FBE, femoral bifurcation endarterectomy; FBE + bypass, femoral bifurcation endarterectomy and additional bypass; BMI: body mass index (calculated as weight in kilograms divided by the square of height in meters). PTA, percutaneous transluminal angioplasty with or without stenting; ABI, ankle brachial index.

**Table 2:** Clinical and angiographic data, GLASS classification, WIfi of wound and foot infection grading, and lesion anatomy of the femoral bifurcation.

	Group A (FBE, *n* = 73)	Group B (FBE + bypass, *n* = 60)	*p*-Value
GLASS femoropopliteal grade			
Grade 3	29 (39.7%)	19 (31.6%)	0.36
Grade 4	44 (60.2%)	41 (68.3%)	0.36
GLASS infrapopliteal grade			
Grade 1	7 (9.6%)	4 (6.7%)	0.75
Grade 2	14 (19.2%)	7 (11.7%)	0.33
Grade 3	33 (45.2%)	37 (61.7%)	0.80
Grade 4	19 (26%)	12 (20%)	0.53
Wound grading in WIfI classification			
Grade 1	17 (23.3%)	13 (21.7%)	0.83
Grade 2	32 (43.8%)	23 (38.3%)	0.59
Grade 3	24 (32.9%)	25 (41.7%)	0.36
Foot infection grading in WIfI classification			
Grade 1	24 (32.9%)	13 (21.7%)	0.17
Grade 2	47 (64.4%)	43 (71.7%)	0.45
Grade 3	2 (2.7%)	4 (6.7%)	0.40
Lesion anatomy of the femoral bifurcation			
CFA isolated	13 (17.8%)	9 (15%)	0.81
CFA + proximal SFA	27 (37%)	24 (40%)	0.72
CFA + DFA	10 (13.7%)	10 (16.7%)	0.63
CFA + DFA + proximal SFA	26 (35.6%)	18 (30%)	0.57
Mean length of CFA lesion (cm ± SD)	2.65 ± 0.85	2.52 ± 0.86	0.12
Mean diameter of treated CFA (mm ± SD)	6.48 ± 0.44	6.3 ± 0.51	0.41
Mean diameter of proximal DFA (mm ± SD)	5.75 ± 0.29	5.37 ± 0.38	0.57

GLASS, Global Limb Anatomic Staging System; WIfI, Wound, Ischemia, and Foot Infection; CFA, common femoral artery; SFA, superficial femoral artery; DFA, deep femoral artery.

## Data Availability

The data presented in this study are available on request from the corresponding author.

## References

[1] Aitken S.J., Randall D.A., Noguchi N., Blyth F.M., Naganathan V. (2020). Multiple peri-operative complications are associated with reduced long term amputation free survival following revascularisation for lower limb peripheral artery disease: A population based linked data study. Eur. J. Vasc. Endovasc. Surg..

[2] Conte M.S., Bradbury A.W., Kolh P., White J.V., Dick F., Fitridge R., Mills J.L., Ricco J.-B., Suresh K.R., Murad M.H. (2019). Global Vascular Guidelines on the Management of Chronic Limb-Threatening Ischemia. Eur. J. Vasc. Endovasc. Surg..

[3] Elsherif M., Tawfick W., Elsharkawi M., Campell R., Hynes N., Sultan S. (2018). Common femoral artery endarterectomy in the age of endovascular therapy. Vascular.

[4] Kuo T.T., Chen P.L., Huang C.Y., Lee C.Y., Shih C.C., Chen I.M. (2019). Outcome of drug-eluting balloon angioplasty versus endarterectomy in common femoral artery occlusive disease. J. Vasc. Surg..

[5] Setacci C., De Donato G., Teraa M., Moll F.L., Ricco J.B., Becker F., Robert-Ebadi H., Cao P., Eckstein H.-H., De Rango P. (2011). Chapter IV: Treatment of critical limb ischaemia. Eur. J. Vasc. Endovasc. Surg..

[6] Kuma S., Tanaka K., Ohmine T., Morisaki K., Kodama A., Guntani A., Ishida M., Okazaki J., Mii S. (2016). Clinical outcome of surgical endarterectomy for common femoral artery occlusive disease. Circ. J..

[7] Nishibe T., Maruno K., Iwahori A., Fujiyoshi T., Suzuki S., Takahashi S., Ogino H., Nishibe M. (2015). The Role of Common Femoral Artery Endarterectomy in the Endovascular Era. Ann. Vasc. Surg..

[8] Langenberg J.C.M., Te Slaa A., De Groot H.G.W., Ho G.H., Veen E.J., Buimer T.M.G., Van der Laan L. (2018). Infection Risk Following Common Femoral Artery Endarterectomy Versus a Hybrid Procedure. Ann. Vasc. Surg..

[9] Soden P.A., Zettervall S.L., Shean K.E., Deery S.E., Kalish J.A., Healey C.T., Kansal N., Schermerhorn M.L. (2017). Effect of adjunct femoral endarterectomy in lower extremity bypass on perioperative and 1-year outcomes. J. Vasc. Surg..

[10] Peters A.S., Meisenbacher K., Weber D., Bisdas T., Torsello G., Böckler D., Bischoff M.S., Collaborators C. (2021). Isolated femoral artery revascularisation with or without iliac inflow improvement—A less invasive surgical option in critical limb ischemia. Vasa.

[11] Malgor R.D., Ricotta J.J., Bower T.C., Oderich G.S., Kalra M., Duncan A.A., Gloviczki P. (2012). Common femoral artery endarterectomy for lower-extremity ischemia: Evaluating the need for additional distal limb revascularization. Ann. Vasc. Surg..

[12] Zlatanovic P., Mahmoud A.A., Cinara I., Cvetic V., Lukic B., Davidovic L. (2021). Comparison of long term outcomes after endovascular treatment versus bypass surgery in chronic limb threatening ischaemia patients with long femoropopliteal lesions. Eur. J. Vasc. Endovasc. Surg..

[13] Jaff M.R., White C.J., Hiatt W.R., Fowkes G.R., Dormandy J., Razavi M., Reekers J., Norgren L. (2015). An update on methods for revascularization and expansion of the TASC lesion classification to include below-the-knee arteries: A supplement to the inter-society consensus for the management of peripheral arterial disease (TASC II): The TASC steering committee. Catheter. Cardiovasc. Interv..

[14] Furuyama T., Onohara T., Yamashita S., Yoshiga R., Yoshiya K., Inoue K., Morisaki K., Kyuragi R., Matsumoto T., Maehara Y. (2018). Prognostic factors of ulcer healing and amputation-free survival in patients with critical limb ischemia. Vascular.

[15] Rutherford R.B., Baker J.D., Ernst C., Johnston K.W., Porter J.M., Ahn S., Jones D.N. (1997). Recommended standards for reports dealing with lower extremity ischemia: Revised version. J. Vasc. Surg..

[16] Ballotta E., Gruppo M., Mazzalai F., Da Giau G. (2010). Common femoral artery endarterectomy for occlusive disease: An 8-year single-center prospective study. Surgery.

[17] Kang J.L., Patel V.I., Conrad M.F., Lamuraglia G.M., Chung T.K., Cambria R.P. (2008). Common femoral artery occlusive disease: Contemporary results following surgical endarterectomy. J. Vasc. Surg..

[18] Chang H., Veith F.J., Rockman C.B., Cayne N.S., Jacobowitz G.R., Garg K. (2022). Non-reversed and reversed great saphenous vein graft configurations offer comparable early outcomes in patients undergoing infrainguinal bypass. Eur. J. Vasc. Endovasc. Surg..

[19] Nierlich P., Enzmann F.K., Metzger P., Dabernig W., Aspalter M., Akhavan F., Hitzl W., Hölzenbein T. (2020). Alternative venous conduits for below knee bypass in the absence of ipsilateral great saphenous vein. Eur. J. Vasc. Endovasc. Surg..

[20] El-Bakr A., Tawfick W., Tubassam M. (2015). Limited common femoral endarterectomy, & profundoplasty as an effective option in limb threatening ischaemia. A minimalistic approach in high-risk patients. Eur. J. Vasc. Endovasc. Surg..

[21] Kim T.I., Zhang Y., Cardella J.A., Guzman R.J., Ochoa Chaar C.I. (2021). Outcomes of bypass and endovascular interventions for advanced femoropopliteal disease in patients with premature peripheral artery disease. J. Vasc. Surg..

[22] De Athayde Soares R., Matielo M.F., Brochado Neto F.C., Martins Cury M.V., Matoso Chacon A.C., Nakamura E.T., Sacilotto R. (2018). The importance of the superficial and profunda femoris arteries in limb salvage following endovascular treatment of chronic aortoiliac occlusive disease. J. Vasc. Surg..

[23] Manenti A., Roncati L., Manco G., Zizzo M., Farinetti A. (2021). Pathophysiology of the profunda femoris artery in chronic lower limb ischemia. Ann. Vasc. Surg..

